# Characterizing the mucin-degrading capacity of the human gut microbiota

**DOI:** 10.1038/s41598-022-11819-z

**Published:** 2022-05-19

**Authors:** Janiece S. Glover, Taylor D. Ticer, Melinda A. Engevik

**Affiliations:** 1 Department of Regenerative Medicine & Cell Biology, Medical University of South Carolina, Charleston, SC USA; 2 Department of Microbiology & Immunology, Medical University of South Carolina, Charleston, SC USA

**Keywords:** Microbial ecology, Microbiome

## Abstract

Mucin-degrading microbes are known to harbor glycosyl hydrolases (GHs) which cleave specific glycan linkages. Although several microbial species have been identified as mucin degraders, there are likely many other members of the healthy gut community with the capacity to degrade mucins. The aim of the present study was to systematically examine the CAZyme mucin-degrading profiles of the human gut microbiota. Within the Verrucomicrobia phylum, all *Akkermansia glycaniphila* and *muciniphila* genomes harbored multiple gene copies of mucin-degrading GHs. The only representative of the Lentisphaerae phylum, *Victivallales*, harbored a GH profile that closely mirrored *Akkermansia*. In the Actinobacteria phylum, we found several *Actinomadura, Actinomyces, Bifidobacterium, Streptacidiphilus* and *Streptomyces* species with mucin-degrading GHs. Within the Bacteroidetes phylum, *Alistipes, Alloprevotella, Bacteroides, Fermenitomonas Parabacteroides, Prevotella* and *Phocaeicola* species had mucin degrading GHs. Firmicutes contained *Abiotrophia, Blautia, Enterococcus, Paenibacillus, Ruminococcus, Streptococcus,* and *Viridibacillus* species with mucin-degrading GHs. Interestingly, far fewer mucin-degrading GHs were observed in the Proteobacteria phylum and were found in *Klebsiella, Mixta, Serratia* and *Enterobacter* species. We confirmed the mucin-degrading capability of 23 representative gut microbes using a chemically defined media lacking glucose supplemented with porcine intestinal mucus. These data greatly expand our knowledge of microbial-mediated mucin degradation within the human gut microbiota.

## Introduction

The intestinal mucus layer is a major component of the boundary region separating the luminal contents from the gut mucosa. Mucus functions as a barrier, a lubricant, an immune cell signal, a reservoir of signaling peptides, and a habitat for indigenous enteric bacteria^1–6^. The intestinal mucus layer is produced by specialized cells known as goblet cells. In the mammalian intestine, goblet cells synthesize and secrete the mucin protein MUC2. The MUC2 protein is decorated with O-glycans, which have core structures of α- and β-linked N-acetyl-glucosamine, N-acetyl-galactosamine, and galactose. These core structures are elongated and commonly modified by α-linked fucose and sialic acid residues^3^. These structurally complex mucin glycans make up approximately 80% of mucin mass. As glycoproteins, mucins can serve as a nutrient source for the resident gut microbes. Bacteria that harbor specific glycosyl hydrolases (GHs) are capable of enzymatically degrading mucin glycans. These released glycan oligosaccharides can then be used as a primary carbohydrate source for the mucus-associated microbiota, providing a sustainable and consistent nutrient supply^2,7^. It has been speculated that the ability to cleave and metabolize mucin O-linked glycans may be an important factor in determining which bacterial species colonize the outer mucus layer.

The degradation of mucin glycans requires the cooperative action of several glycsyl hydrolases encoded by the genomes of mucin-degrading bacteria^2,3,8,9^. To access mucin glycans, intestinal microbes must express the GH33 sialidases (also known as neuraminidases), which cleave terminal sialic acid residues. Microbes may also produce GH29 or GH95 to remove fucose residues. Following the removal of terminal sugars, bacteria can harbor N-acetyl-glucosaminidases (GH84, GH85, G89, GH20), N-acetyl-galactosaminidases (GH101, GH129), and galactosidases (GH2, GH35, GH42, GH98). There are also endo-acting O-glycanases (GH16) which can cleave large glycan structures. Known mucin-degrading bacterial strains include *Akkermansia muciniphila*, *Bacteroides* spp., *Bifidobacterium* spp., *Ruminococcus* spp., *Clostridium* spp., *Paraclostridium* spp. and *Prevotella* spp.^3,9–24^. The best studied mucin degrading microbes are *Akkermansia muciniphila* and *Bacteroides* spp. *Akkermansia muciniphila* is considered to be a mucin-specialist, as it can employ several enzyme combinations to hydrolyze up to 85% of mucin structures^25^. *Bacteroides* spp. are general glycan degraders and certain *Bacteroides* spp. are able to switch from dietary glycans to mucin glycans due to their extensive arrays of carbohydrate-active enzymes^2^. For example, *B. thetaiotaomicron* can extensively degrade mucin glycans and forage in the mucus layer when plant polysaccharides are absent from the diet^11,26^. Despite the growing number of bacterial genome sequences available, our knowledge of the mucin-degrading capacity of other microbes, particularly commensal human gut microbes, remains fragmented. The aim of the present study was to systematically examine the CAZyme mucin-degrading profiles of the human gut microbiota.

## Methods

### Bacterial culturing

The following strains were grown anaerobically at 37 °C in brain-heart-infusion (BHI) supplemented with 2% yeast extract and 0.2% cysteine: *Bacteroides vulgatus* ATCC 8482, *Bacteroides thetaiotaomicron* ATCC 29148, *Bacteroides fragilis* MGH 10513, *Blautia coccoides* ATCC 29236, *Blautia producta* ATCC 27340D, *Parabacteroides merdae* MGH 10511, *Clostridium butyricum* CB, *Clostridium symbiosum* ATCC 14940, *Clostridium inoculum* ATCC 14501, *Clostridium clostridiforme* ATCC 25532, *Clostridium sporogenes* DSMZ 795, and *Prevotella copri* DSZM 18205. *Akkermansia muciniphila* ATCC BAA-835 was grown in BHI supplemented with 2% yeast extract, 0.2% cysteine, and 0.4% porcine gastric mucin (Sigma). The following strains were grown anaerobically at 37 °C in Man-DeRosa-Sharp (MRS): *Lactobacillus gasseri* ATCC 33323, *Lactobacillus johnsonii* ATCC 33200, *Lactobacillus brevis* ATCC 27305, *Lactobacillus acidophilus* ATCC 4796, *Bifidobacterium dentium* ATCC 27678, *Bifidobacterium longum* subsp. *infantis* ATCC 15697, *Bifidobacterium bifidum* ATCC 29521, *Bifidobacterium longum* ATCC 55813, and *Bifidobacterium angulatum* ATCC 27535. All cultures were grown in an Anaerobe Systems AS-150 anaerobic chamber supplied with a mixture of 10% CO_2_, 5% H_2_, and 85% N_2_. *E. coli* Nissle 1917 was grown aerobically at 37 °C in LB broth.

To assess mucin-degradation, overnight cultures were centrifuged at 6000×*g* for 5 min to pellet the bacteria and the bacterial pellet was washed 3× to remove traces of the rich media. After the final wash, the bacterial pellet was resuspended in an equal volume of a chemically defined culture medium ZMB1^27^ lacking glucose and sub-cultured to an optical density (OD_600nm_) of 0.1. The culture conditions included: (1) ZMB1 lacking glucose, (2) ZMB1 with 100 mM Glucose or (3) ZMB1 lacking glucose with 1 mg/mL pig intestinal mucin (MyBiosource cat# MBS2028824 > 90% purity, dialyzed in water with SnakeSkin™ Dialysis Tubing, 10 K MWCO, FisherSci #P168100). All cultures were grown anaerobically at 37 °C and growth was monitored by measuring OD_600nm_ after 20 hours of incubation.

### Computational analysis

The glycosyl hydrolase (GH) families involved in mucin degradation were downloaded from the Carbohydrate-Active enZYmes (CAZy) database (https://www.cazy.org) and examined as previously described^28–33^. Gene copy numbers were collected from all annotated genomes. The glycosyl hydrolases known to be involved in mucin degradation (GH33, 16, 29, 95, 20, 2, 35, 42, 98, 101, 129, 89, 85, and 84) were included for analysis. Of the 20,954 genomes available in the CAZy database, we identified 13,156 genomes harboring at least one gene copy of at least one GH family involved in mucin degradation. Microbes from healthy individuals were identified in the Human Microbiome Project (HMP) using the Integrated Microbial Genomes (IMG) database (img.jgi.doe.gov) available through the Joint Genomes Institute (JGI) (Version 6.0)^34^. Removal of non-gut microbes and microbes not identified in healthy individuals resulted in 4385 genomes for downstream analysis. Any microbes in question were further examined by a literature search.

### Statistics

GraphPad Prism (version 9) software (GraphPad Inc., La Jolla, CA) was used for all statistics. Growth was examined using a one-way analysis of variance (ANOVA). Differences between the groups were considered significant at P < 0.05 (*).

## Results

**Figure 1:**
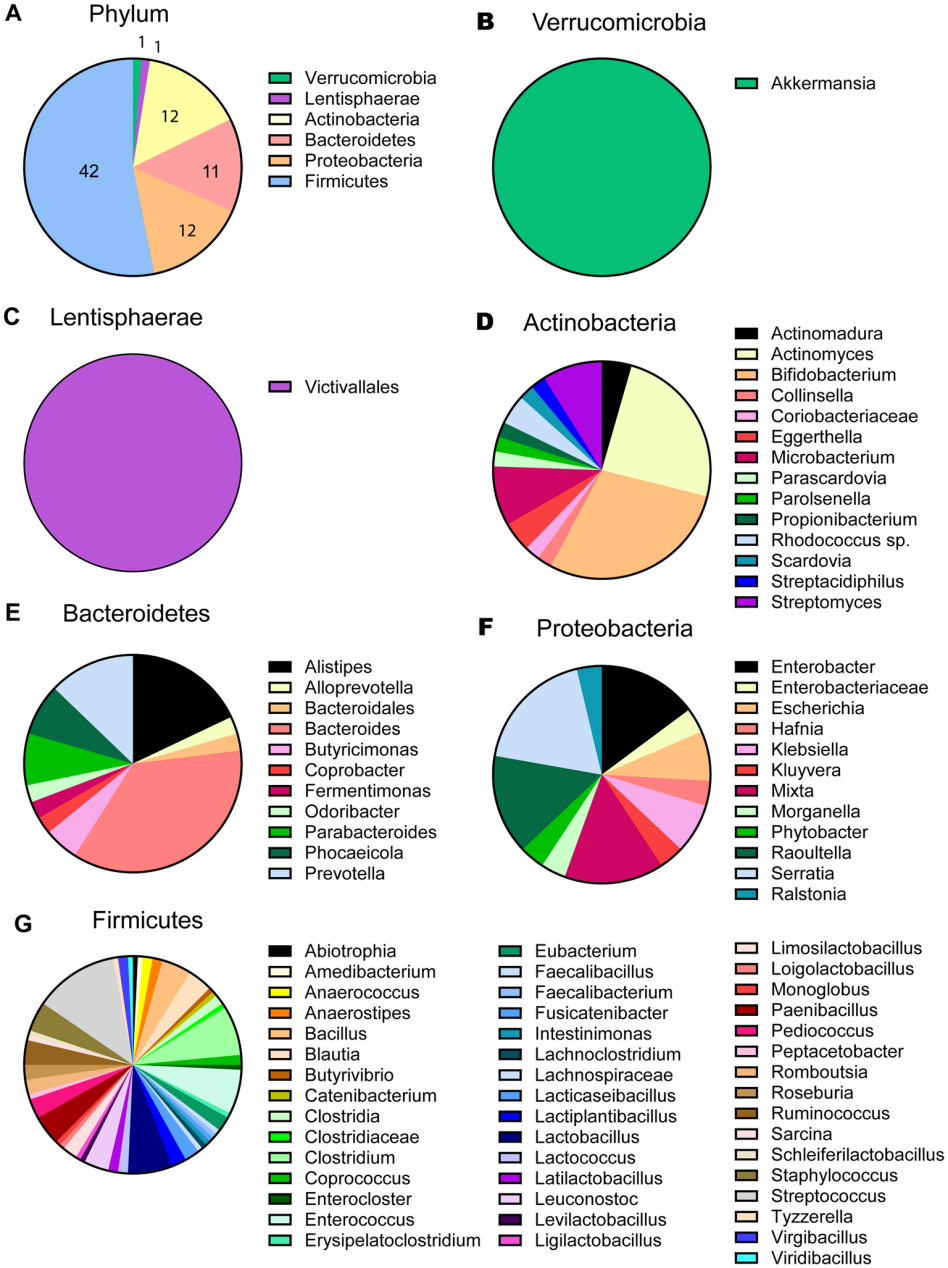
Human gut microbes harboring mucin associated glycosyl hydrolases (GHs) are well distributed among the bacterial phyla. (**A**) Distribution of genera within each bacteria phlya that possess mucin-related GHs. Distribution of genera within (**B**) Verrucomicrobia, (**C**) Lentisphaerae, (**D**) Actinobacteria, (**E**) Bacteroidetes, (**F**) Proteobacteria and (**G**) Firmicutes phlya that harbor at least one mucin-related GH.

We identified 4385 human gut microbial genomes harboring at least one gene copy of a mucin-degrading GH family. We found one genus in the Verrucomicrobia and Lentisphaerae phyla, 12 different genera in the Actinobacteria and Proteobacteria phyla, 11 genera in Bacteroidetes, and 42 genera in the Firmicutes phylum (Fig. 1A). In the Verrucomicrobia phylum, *Akkermansia* was the sole genus (Fig. 1B). Likewise, within the Lentisphaerae phylum, *Victavallales* was the only genus (Fig. 1C). In the Actinobacteria phylum, *Bifidobacterium* had the highest representation, with 13 *Bifidobacteria* genera identified, followed closely by *Actinomyces* (11 genera), *Microbacterium* (4 genera), and *Streptomyces* (4 genera) (Fig. 1D). In the Bacteroidetes phylum, we observed high representation of *Bacteroides* (14 genera), *Alistipes* (7) and *Prevotella* (5) (Fig. 1E). Within Proteobacteria, we observed *Serratia* (5 genera), *Raoultella* (4), *Mixta* (4) and *Enterobacter* (4) at relatively similar levels (Fig. 1F). Multiple genera were identified in the Firmicutes phylum, with the most abundant microbes being *Streptococcus* (17 genera), *Clostridium* (10), *Enterococcus* (9), *Lactobacillus* (9), *Bacillus* (6), *Paenibacillus* (6), *Staphylococcus* (6), *Blautia* (5), *Ruminococcus* (5), among others (Fig. 1G).

**Figure 2:**
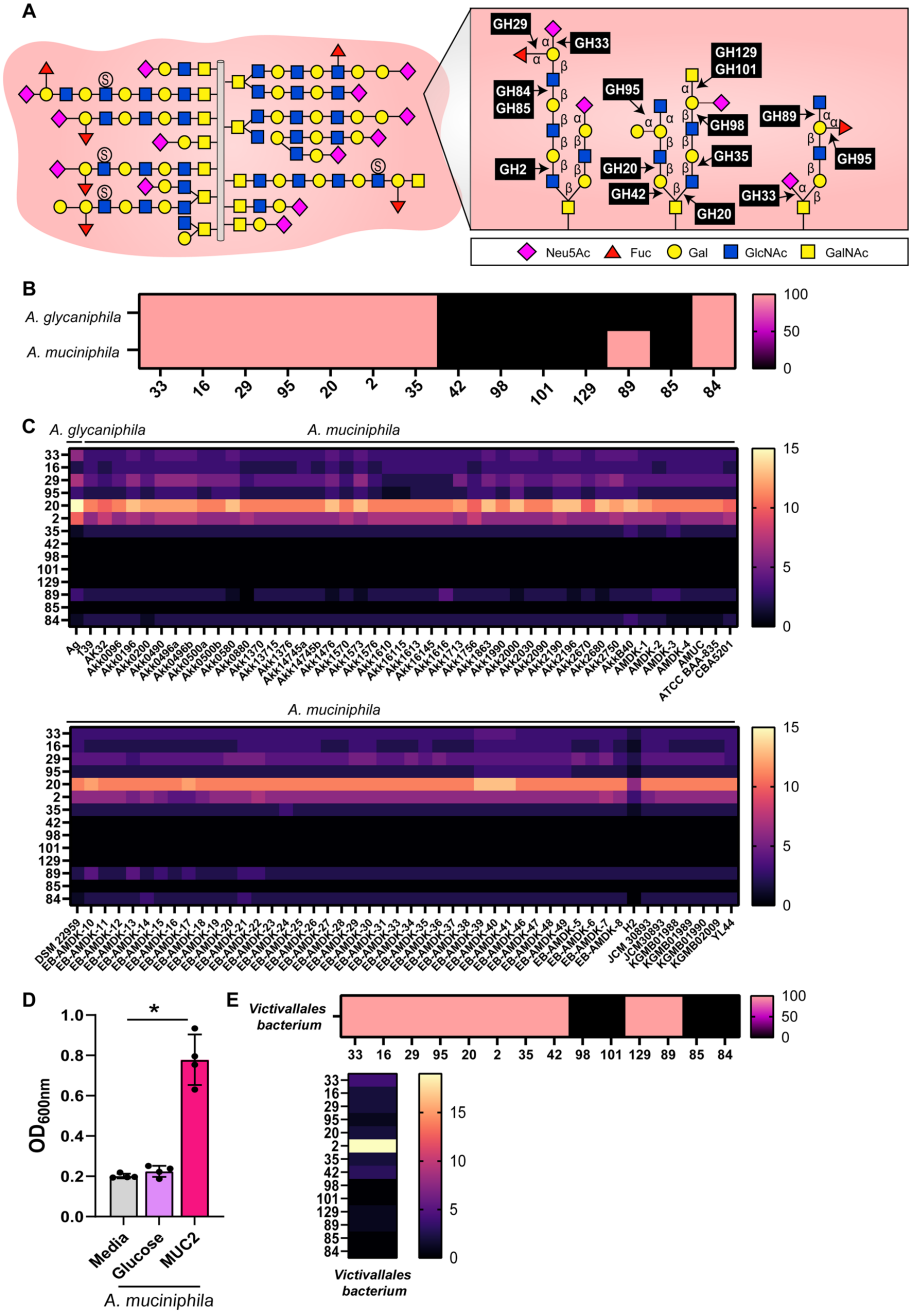
Mucin-related glycosyl hydrolase profiles in the Verrucomicrobia and Lentisphaerae phlyum. (**A**) Representative intestinal mucin glycans structures and corresponding microbial GHs. (**B**) Heat map of the percentage of *Akkermansia glycaniphila* or *Akkermansia muciniphila* genomes that have at least one gene copy of mucin-associated GH mucin-associated GH 33, 16, 29, 95, 20, 2, 35, 42, 98, 101, 129, 89, 85, and 84. (**C**) Heat maps depicting the gene copy number of mucin-associated GHs in the strains of *A. glycaniphila* and A. *muciniphila*. (**D**) Growth analysis of *A. muciniphila* ATCC BAA-835 in a chemically defined media ZMB1 lacking glucose (media control), with glucose (positive control), or lacking glucose and supplemented with 1 mg/mL porcine intestinal MUC2. Growth was measured by examining the optical density at 600 nm (OD_600nm_) after overnight incubation. (**E**) Heat maps showing the percentage of genomes that have at least one gene copy of each mucin-associated GH and depicting the gene copy number of mucin-associated GHs in the one strain of *Victivallales bacterium*.

To access mucin glycans, intestinal microbes must possess mucin-degrading glycosyl hydrolases (Fig. 2A)^2^. Released mucin glycan oligosaccharides can then be used to support the growth of bacteria. Given the prominence of *Akkermansia* as a mucin-degrading genus, we first analyzed the genomes of human gut microbes *A. glycaniphila* and *A. muciniphila* (Fig. 2B). The one available genome of *A. glycaniphila* contained a least one gene copy of GH33 (sialidase), GH16 (endo-acting O-glycanase), GH29 (fucosidase), GH95 (fucosidase), GH20 (galactosidase), GH2 (galactosidase), GH35 (galactosidase), and GH84 (N-acetyl-glucosaminidases). Similarly, all the *A. muciniphila* genomes contained a least one gene copy of GH33, GH16, GH29, GH95, GH20, GH2, GH35 and GH84, as well as GH89, indicating that *A. muciniphila* can cleave sialic acid, fucose, galactose, and N-acetylglucosamine. Closer examination of the *Akkermansia* genomes revealed that the one genome of *A. glycaniphila* had six gene copes of GH33 and all 95 of the *A. muciniphila* genomes contained 2–4 genes copies of GH33 (Fig. 2C), indicating that *Akkermansia* spp. have the capacity to remove sialic acid and initiate mucin-degradation. The GHs with the largest gene copy range (6–13 gene copies) was GH20, a family containing β-N-acetyl-glucosaminidases (Table 1). No *Akkermansia* genomes contained GH42, 98, 101, 129 or 85, suggesting that *Akkermansia* is unable to degrade N-acetyl-galactosamine. To confirm the capacity of *A. muciniphila* to degrade intestinal mucus, we grew *A. muciniphila* ATCC BAA-835 is a chemically defined media ZMB1 lacking glucose, containing 100 mM glucose or containing 1 mg/mL porcine intestinal mucus (Fig. 2D). As expected, *A. muciniphila* had limited growth in ZMB1 with or without glucose but exhibited robust growth in media with porcine intestinal mucus. These findings complement our genome analysis of *A. muciniphila* ATCC BAA-835 (the BAA-835 genome analysis is found in the second column from the right in Fig. 2C). Additionally, various *A. muciniphila* strains were also examined to showcase the diversity of GH profiles across the genus, which supports the ability of this species to degrade mucins.

**Table 1:** Mucin-associated glycosyl hydrolase (GH) families with corresponding enzyme commission numbers and description.

GH family	Enzyme commission #	Description
2	EC 3.2.1.23	β-galactosidase
	EC 3.2.1.25	β-mannosidase
	EC 3.2.1.31	β-glucuronidase
	EC 3.2.1.55	α-l-arabinofuranosidase
	EC 3.2.1.152	Mannosylglycoprotein endo-β-mannosidase
	EC 3.2.1.165	Exo-β-glucosaminidase
	EC 3.2.1.-	α-l-arabinopyranosidase
	EC 3.2.1.-	β-galacturonidase
	EC 3.2.1.37	β-xylosidase
	EC 3.2.1.146	β-d-galactofuranosidase
	EC 3.2.1.21	β-glucosidase
16	EC 2.4.1.207	Xyloglucan:xyloglucosyltransferase
	EC 3.2.1.103	Keratan-sulfate endo-1,4-β-galactosidase
	EC 3.2.1.39	Endo-1,3-β-glucanase/laminarinase
	EC 3.2.1.6	Endo-1,3(4)-β-glucanase
	EC 3.2.1.73	Licheninase
	EC 3.2.1.81	β-agarase
	EC 3.2.1.83	κ-carrageenase
	EC 3.2.1.151	Xyloglucanase
	EC 3.2.1.181	Endo-β-1,3-galactanase
	EC 3.2.1.178	β-porphyranase
	EC 3.2.1.35	Hyaluronidase
	EC 3.2.1.-	Endo-β-1,4-galactosidase
	EC 2.4.1.-	Chitin β-1,6-glucanosyltransferase
	EC 2.4.1.-	β-transglycosidase
	EC 3.2.1.-	β-glycosidase
	EC 3.2.1.-	β-carrageenase
20	EC 3.2.1.52	β-hexosaminidase
	EC 3.2.1.140	Lacto-N-biosidase
	EC 3.2.1.-	β-1,6-N-acetylglucosaminidase
	EC 3.2.1.-	β-6-SO_3_-N-acetylglucosaminidase
29	EC 3.2.1.51	α-l-fucosidase
	EC 3.2.1.111	α-1,3/1,4-l-fucosidase
	EC 3.2.1.63	α-1,2-l-fucosidase
33	EC 3.2.1.18	Sialidase or neuraminidase
	EC 2.4.1.-	Trans-sialidase
	EC 4.2.2.15	Anhydrosialidase
	EC 3.2.1.-	Kdo hydrolase
	EC 3.2.1.-	2-Keto-3-deoxynononic acid hydrolase/KDNase
35	EC 3.2.1.23	β-galactosidase
	EC 3.2.1.165	Exo-β-glucosaminidase
	EC 3.2.1.-	Exo-β-1,4-galactanase
	EC 3.2.1.-	β-1,3-galactosidase
42	EC 3.2.1.23	β-galactosidase
	EC 3.2.1.-	α-l-arabinopyranosidase
84	EC 3.2.1.52	N-acetyl β-glucosaminidase
	EC 3.2.1.35	Hyaluronidase
	EC 3.2.1.169	[Protein]-3-O-(GlcNAc)-l-Ser/Thr β-N-acetylglucosaminidase
85	EC 3.2.1.96	Endo-β-N-acetylglucosaminidase
89	EC 3.2.1.50	α-N-acetylglucosaminidase
95	EC 3.2.1.51	α-l-fucosidase
	EC 3.2.1.63	α-1,2-l-fucosidase
	EC 3.2.1.-	α-l-galactosidase
98	EC 3.2.1.102	Endo-β-1,4-galactosidase
	EC 3.2.1.-	Endo-β-1,4-galactosidase
	EC 3.2.1.8	endo-β-1,4-xylanase
101	EC 3.2.1.97	Endo-α-N-acetylgalactosaminidase
129	EC 3.2.1.49	α-N-acetylgalactosaminidase
	EC 3.2.1.-	α-1,3-(3,6)-anhydro-d-galactosidase

Next, we examined *Victavallales bacterium* in the Lentisphaerae phylum (Fig. 2E). Genome analysis revealed a similar GH profile to *Akkermansia*, with genes for GH33, GH16, GH29, GH95, GH20, GH2, GH35, and GH89, suggesting that *Victavallales bacterium* could enzymatically cleave sialic acid, fucose, galactose, and N-acetyl-glucosamine. Interestingly, *Victavallales* also harbored gene copies of GH42 and GH129, GHs not found in *Akkermansia*. The presence of GH129 indicates that *Victavallales bacterium* can release N-acetyl-galactosamine, a glycan which *Akkermansia* is not able to cleave. *Victavallales bacterium* possessed 4 genes copies of GH33 and 19 gene copies of GH2, which contains β-galactosidases. Although little information is available for *Victavallales bacterium*, the genome analysis reveals that *Victavallales bacterium* could degrade mucins.

**Figure 3:**
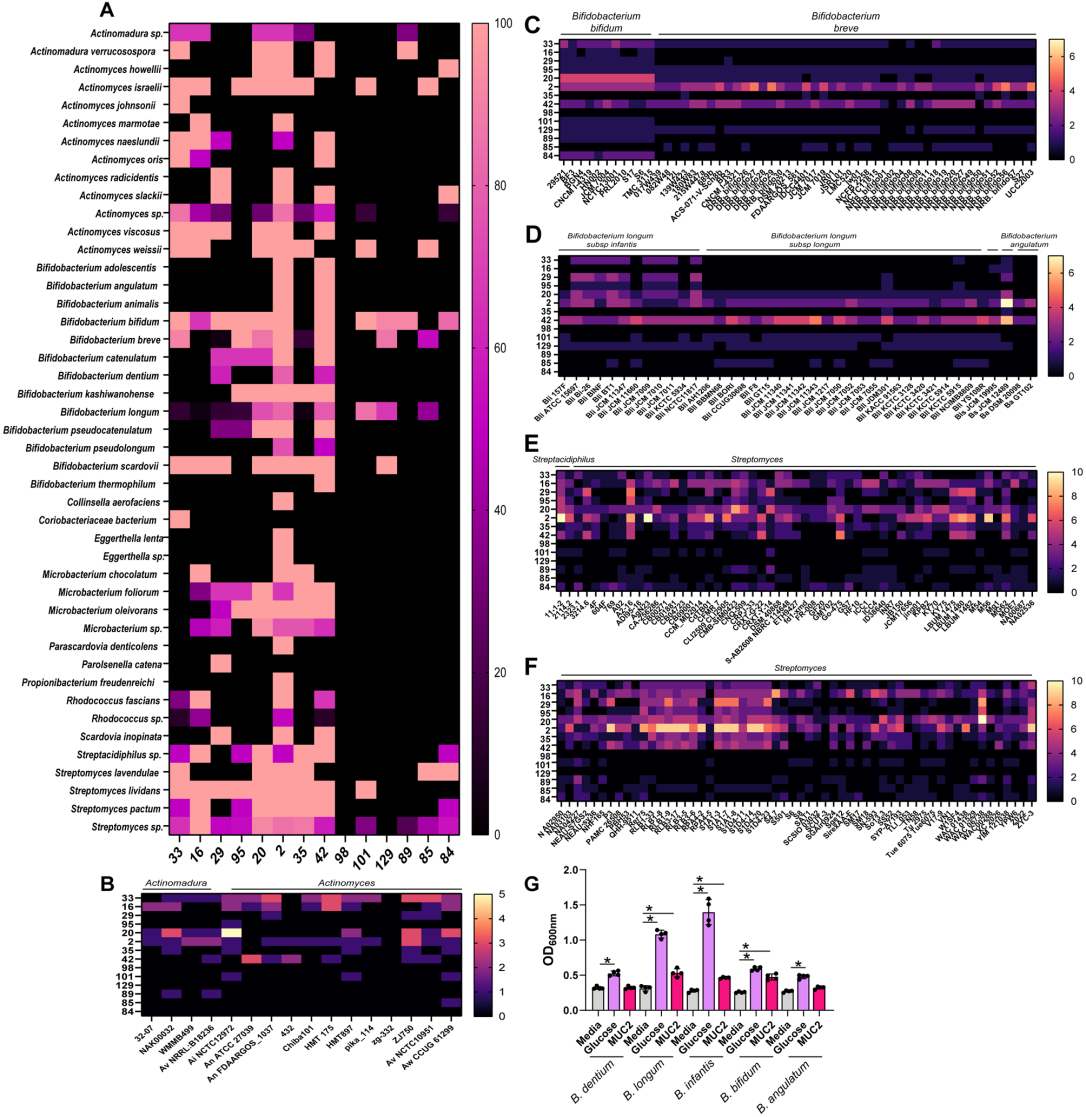
Mucin-related glycosyl hydrolase profiles in the Actinobacteria phlyum. (**A**) Heat map of the Actinobacteria genomes that have at least one gene copy of a mucin-associated GH 33, 16, 29, 95, 20, 2, 35, 42, 98, 101, 129, 89, 85, and 84. (**B**) Heat map showing the gene copy number of mucin-associated GHs in the strains of *Actinomadura* and *Actinomyces,* (**C**) *Bifidobacteria,* specifically *B. bifidum* and *B. breve,* (**D**) *B. longum* (Bl), *B. longum* subsp*. infantis* (Bli)*, B. longum* subsp*. longum* (Bll)*, B. longum* subsp*. suillum* (Bls), and *B. scardovii*, (**E**) *Streptacidiphilus and Streptomyces* species, and (**F**) *Streptomyces* species. (**G**) Growth analysis of *Bifidobacterium dentium* ATCC 27678, *B. longum* subsp. *infantis* ATCC 15697, *B. bifidum* ATCC 29521, *B. longum* ATCC 55813, and *B. angulatum* ATCC 27535 in a chemically defined media ZMB1 lacking glucose (media control), with glucose (positive control), or lacking glucose and supplemented with 1 mg/mL porcine intestinal MUC2. Growth was measured by examining the optical density at 600 nm (OD_600nm_) after overnight incubation.

Within the Actinobacterium phylum, we identified several genera harboring mucin-degrading GHs, including *Actinomadura, Actinomyces, Bifidobacteria, Streptacidiphilus* and *Streptomyces* species (Fig. 3A). We observed that 3 of the 4 *Actinomadura* spp. had 1 gene copy of GH33, as well as gene copies of GH16, GH20, GH2, GH35, GH84 and GH89, suggesting the ability of *Actinomadura* spp. to remove sialic acid, galactose and N-acetyl-glucosamine (Fig. 3B). We found that all the genomes of *Actinomyces israelii, A. naeslundii, A. viscosus* and *A. weissii,* as well as 5 of the 7 genomes of undefined *Actinomyces* spp. contained 2–3 gene copies of GH33. Actinomyces members also contained gene copies for GH16, GH29, GH20, GH2, GH35, GH42, and GH101. This glycosyl hydrolase profile indicates the *Actinomyces* spp. can potentially cleave all mucin glycans.

In *Bifidobacterium* (Fig. 3C), we found that all 11 genomes of *B. bifidum* had 1–3 gene copies of GH33 and all genomes had GH29, GH95, GH20, GH2, GH42, GH101, GH129, GH89 and GH84. Additionally, 8 of the 11 *B. bifidum* genomes had one gene copy of GH16. These GH profiles are consistent with previous studies which identify *B. bifidum* as a mucin degrading microbe since it can remove all mucin glycans^20,22^. Within the 44 *B. breve* genomes, we found that 41 genomes had one gene copy of GH33 and the majority of strains had GH95, GH20, GH2, GH42, and GH129, covering all mucin glycan structures (Fig. 3C). *B. longum* had much more variability in terms of mucin-degrading GHs (Fig. 3D). Only 11 of the 54 genomes contained GH33, the majority of which belonged to the *B. longum* subspecies *infantis* subgroup. Variable presence for GH29, GH95, GH20, GH2, GH42, GH101, GH129 and GH85 was identified, with genomes harboring 0–5 gene copies. In contrast, *B. angulatum* only possessed 2 mucin-associated GHs: GH2 and GH42, suggesting that this species is likely unable to extensively degrade mucins. These data indicate that mucin degradation is species dependent in Bifidobacteria.

Within the two genomes of *Streptacidiphilus* spp. (Fig. 3E), we found that one of the two genomes had one gene copy of GH33 (sialidase), but both genomes had 10 gene copies of GH16 (endo-O-glycanase) as well as the genes for GH20, GH35, and GH42 (galactosidases). Commensal *Streptomyces lavendulae*, *S. lividans*, and *S. pactum* genomes contained GH33, GH16, GH95, GH2, GH35, GH42, and variable presence of GH101, 89 and 84. Among the 113 undefined *Streptomyces* spp. genomes (Fig. 3E,F), we found that 80 genomes had 1–5 gene copies of GH33, and the majority of strains had gene copies for GH16, GH29, GH95, GH20, GH2, GH35, GH42 and GH84. *Streptomyces* spp. had several copies of GH2, with some strains possessing 10 gene copies. These data suggest that *Streptomyces* species are well adapted to remove sialic acid, fucose, galactose, and N-acetyl-glucosamine.

To confirm our genome findings, we also examined the growth of key Bifidobacteria in ZMB1 with or without glucose or intestinal mucus (Fig. 3G). Our genome analysis revealed that *B. dentium* ATCC 27678 and *B. angulatum* ATCC 27535 did not possess GH33 and had only 2–3 mucin-associated GHs, while *B. longum* and *B. bifidum* had several gene copies of GH33 and other mucin-degrading GHs. In our growth analysis, we did not detect growth above the ZMB1 media baseline when intestinal mucus was added, indicating that these species cannot degrade intestinal mucus to use as a carbon source. In contrast, *B. longum* subsp. *infantis* ATCC 15697, *B. longum* ATCC 55813, and *B. bifidum* ATCC 29521 had enhanced growth when mucus was present, indicating that these strains can degrade mucins.

**Figure 4:**
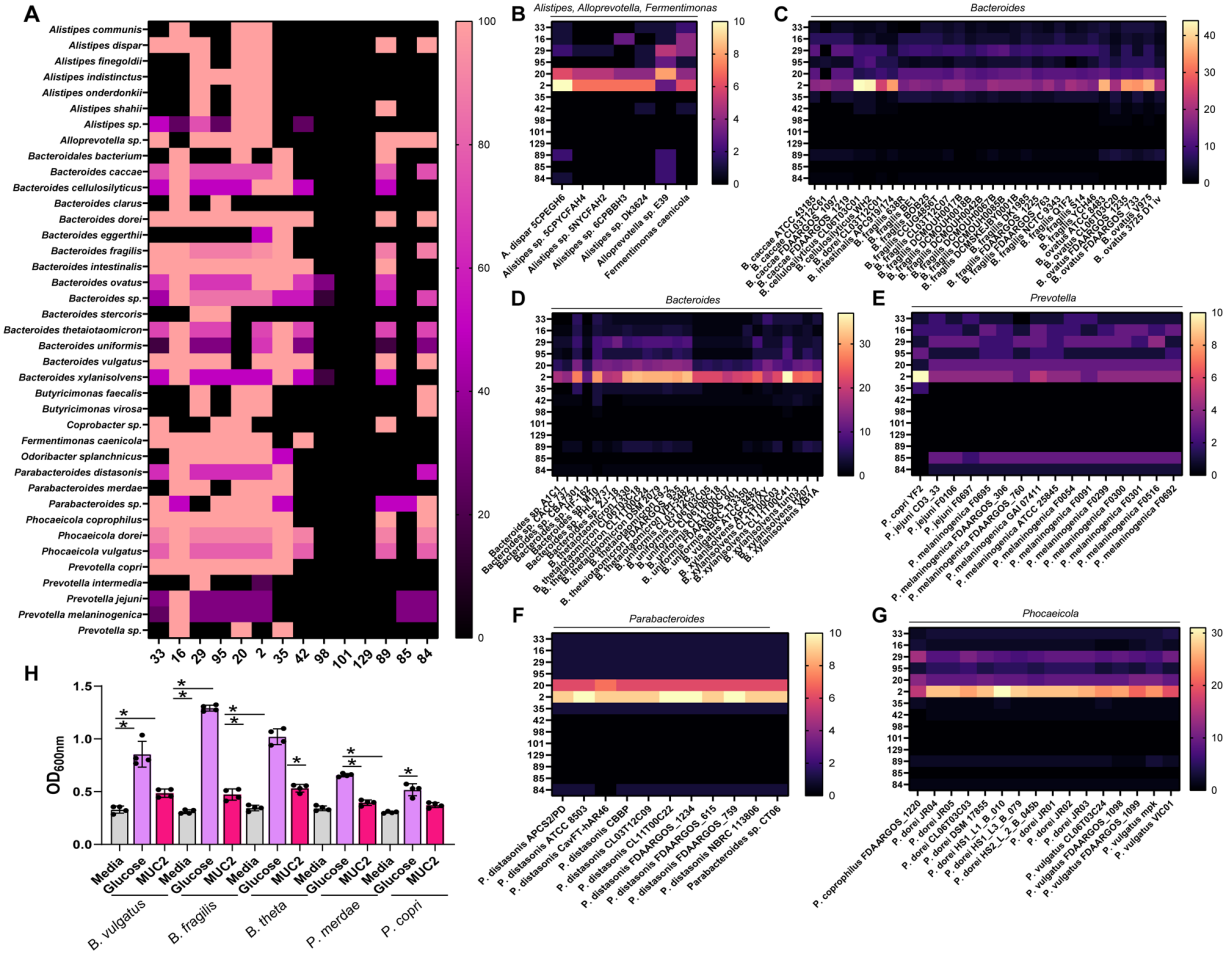
Mucin-related glycosyl hydrolase profiles in the Bacteroidetes phlyum. (**A**) Heat map of the genera within the Bacteroidetes phlyum that have at least one gene copy of each mucin-associated GH 33, 16, 29, 95, 20, 2, 35, 42, 98, 101, 129, 89, 85, and 84. Heat map showing the gene copy number of mucin-associated GHs in the strains of (**B**) *Alistipes, Alloprevotella,* and *Fermenitomonas,* (**C**) *Bacteroides*, specifically *B. caccae, B. dorei, B. intestinalis, B. fragilis and B. ovatus,* (**D**) *Bacteroides,* specifically *Bacteroides* spp., *B. thetaiotaomicron, B. uniformis,* and *B. xylanisolvens,* (**E**) *Prevotella copri, P. jejuni,* and *P melaninogenica,* (**F**) *Parabacteroides and P. distasonis, and* (**G**) *Phocaeicola coprophilus, P. dorei,* and *P. vulgatus.* (**H**) Growth analysis of *Bacteroides vulgatus* ATCC 8482*, B. thetaiotaomicron* ATCC 29148*, B. fragilis* MGH 10513*, Prevotella merdae MGH 10511, and Prevotella copri* DSMZ 18205 in a chemically defined media ZMB1 lacking glucose (media control), with glucose (positive control), or lacking glucose and supplemented with 1 mg/mL porcine intestinal MUC2. Growth was measured by examining the optical density at 600 nm (OD_600nm_) after overnight incubation.

Analysis of genomes within the Bacteroidetes phylum revealed mucin degrading GHs in *Alistipes, Alloprevotella, Bacteroides, Fermentimonas, Parabacteroides, Prevotella* and *Phocaeicola* species (Fig. 4A). Only 2 of the 5 *Alistipes* spp. genomes had one gene copy of GH33, but all genomes had GH20 and GH2 (galactosidase) and most genomes had GH16 (endo-O-glycanase) and GH29 (fucosidase) (Fig. 4B). The one genome of *Alloprevotella* had GH33, GH16, GH29, GH95, GH20, GH2, GH89, GH85 and GH84, one more GH family than *Akkermansia*, potentially indicating that this microbe could be a mucin-degrader. The one genome of *Fermentimonas caenicola* also had gene copies of GH33, GH16, GH29, GH95, GH20, GH2, and GH42. Although there are few reports on this microbe, the GH profiles suggest that this *Fermentimonas caenicola* could also be a mucin degrader. Consistent with the literature, we found a large repertoire of GHs involved in mucin degradation in the *Bacteroides* spp. genomes (Fig. 4C). We found that 3 of the 4 *B. caccae* genomes had 2–3 gene copies of GH33 and all genomes had gene copies of GH16 (endo-O-glycanase), GH95 (fucosidase), GH2 (galactosidase), and 84 (N-acetyl-glucosaminidases). Additionally, 3 of the 4 genomes had gene copies for GH29, GH20, and GH35 families. All of the two *B. cellulosilyticus* genomes harbored GH33, GH16, GH29, GH95, GH20, GH2, GH35, GH42 and GH89. Similar to *B. cellulosilyticus*, all six of the *B. ovatus* genomes, the one *B. dorei* and *B. intestinalis* genome and all 7 of the *B. thetaiotaomicron* genomes had GH33, GH16, GH29, GH95, GH20, GH2, GH35, GH42, and GH89 gene copies. The *B. thetaiotaomicron* genomes also possessed GH84. *B. fragilis* had 16 of the 18 genomes with gene copies for GH33, but all *B. fragilis* genomes harbored GH16, GH29, GH20, GH2, GH35, GH89 and GH84. Additionally, 17 of 18 of the genomes also had GH95. We also examined 7 undefined *Bacteroides* spp., and found gene copies of GH33, GH16, GH29, GH29, GH95, GH20, GH2, and GH84 (Fig. 4D). All *B. uniformis* members also had 1 gene copy of GH42 and *B. vulgatus* had 1 gene copy of GH42 and GH89. *B. xylanisolvens* genomes mirrored the other *Bacteroides* spp., with all 6 genomes harboring GH33, GH16, GH29, GH95, GH20, GH2, GH45, GH42 and 5 of the 6 genomes containing GH89. These data support the notion that many *Bacteroides* members are mucin-degraders.

Within the 16 *Prevotella* genomes (Fig. 4E), we found that the one *P. copri* and three *P. jejuni* genomes all had genes copies for GH33, GH16, GH29, GH85, GH20, and GH2. *P. jejuni* also had 1–3 gene copies of GH85 and GH84. Furthermore, in the 12 *P. melaninogenica* genomes we found that 11 of the 12 had GH33 genes, while all the *P. melaninogenica* genomes had 1–4 gene copies of GH16, GH29, GH95, GH20, GH2, GH85, and GH84. These data indicate that most *P. melaninogenica* are well adapted to cleave sialic acid, fucose, galactose, and N-acetyl-glucosamine. The *Parabacteroides* genomes mirrored the *Prevotella* spp. mucin-degrading GHs, with all genomes harboring GH33, GH16, GH29, GH95, GH20, GH2, GH35 and GH84 (Fig. 4F). In addition, within Bacteroidetes we found that all the 16 *Phocaeicola* spp. genomes contained genes for GH33, GH16, GH29, GH95, GH20, GH2, GH35, GH89 and GH84. We observed that 15 of the 16 *Phocaeicola* spp. genomes also had one gene copy of GH42 (Fig. 4G). We observed multiple gene copies of GH2 in Bacteroidetes. *B. cellulosilyticus* possessed the most GH2 gene copies, with 44 gene copies in total. To confirm the mucin-degrading capacity of *B. vulgatus, B. fragilis*, and *B. thetaiotaomicron*, we grew *B. vulgatus* ATCC 8482, *B. fragilis* MGH 10513 and *B. thetaiotaomicron* ATCC 29148 in ZMB1 with or without glucose or intestinal mucus (Fig. 4H). As expected, each of these microbes grew in ZMB1 lacking glucose supplemented with intestinal mucus, consistent with previous literature showing these microbes can degrade mucins^13,14^. We also grew *Prevotella copri* DSMZ 18205 and *Parabacteroides merdae* MGH 10511 to assess the ability of these *Prevotella* strains to grow in the presence of mucus. *P. merdae* did not grow in mucus, which was consistent with our analysis which showed no gene copies of GH33. Interestingly, although the one genome of *P. copri* in our analysis had GH33 expression and other mucin-degrading GHs, our *P. copri* DSMZ 18205 strain did not grow in ZMB1 lacking glucose with mucus, suggesting that growth might be strain specific.

**Figure 5:**
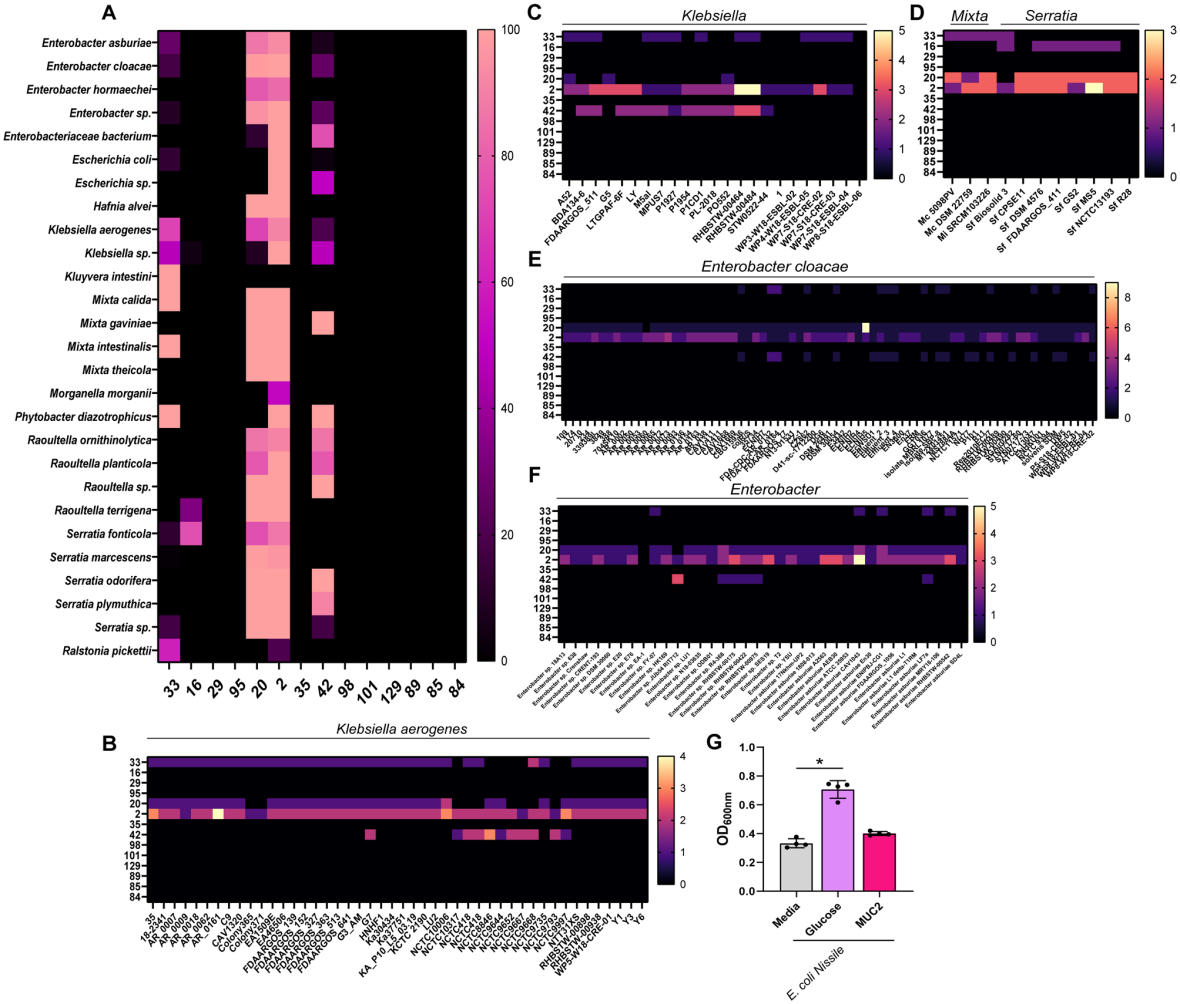
Mucin-related glycosyl hydrolase profiles in the Proteobacteria phlyum. (**A**) Heat map of the Proteobacteria genomes that have at least one gene copy of each mucin-associated GH 33, 16, 29, 95, 20, 2, 35, 42, 98, 101, 129, 89, 85, and 84. Heat map showing the gene copy number of mucin-associated GHs in the strains of (**B**) *Klebsiella aerogenes*, (**C**) *Klebsiella* spp., (**D**) *Mixta calida, M. intestinalis, and Serratia fonticola,* (**E**) *Enterobacter cloacae*, (**F**) *Enterobacter* spp. and *E. asburiae.* (**G**) Growth analysis of *E. coli* Nissle 1917 in a chemically defined media ZMB1 lacking glucose (media control), with glucose (positive control), or lacking glucose and supplemented with 1 mg/mL porcine intestinal MUC2. Growth was measured by examining the optical density at 600 nm (OD_600nm_) after overnight incubation.

Compared to the mucin-degrading microbes identified in other phyla, we observed far fewer mucin-degrading GHs in the Proteobacteria phylum, with only 3–4 GHs families found in *Klebsiella, Mixta* and *Enterobacter* spp. (Fig. 5A). All 46 of the *Klebsiella aerogenes* genomes had one gene copy of GH33 (sialidase) and 1–2 gene copies of GH2 (galactosidase). Ten of the 46 *K. aerogenes* genomes also had expression of GH42 (galactosidase) and 41 of the genomes had 1–2 gene copies of GH20 (galactosidase), suggesting the ability of these strains to remove galactose residues (Fig. 5B). Similarly, all 23 undefined *Klebsiella* spp. genomes had 1–3 gene copies of GH2, but only 13 of the 23 genomes had GH33, 14 genomes had GH42 genes and 3 of the genomes had GH20 (Fig. 5C). No other mucin-degrading GHs genes were observed. Of the four *Mixta* spp., which includes *M. calida* and *M. intestinalis*, we found that all three genomes had 1–2 gene copies of GH33, GH20 and GH2, but no other mucin-related GHs were identified (Fig. 5D). We observed large variation in the 8 *Serratia fonticola* genomes. Only one of the genomes had GH33, 6 genomes had GH16, 7 genomes had GH20 and all 8 genomes had GH2 gene copies. In the *Enterobacter* genera, only 15 of the 73 *E. cloacae* genomes had one gene copy of GH33, although most of the strains had GH20 and GH2 gene copies (Fig. 5E). Similarly, only 5 of the 36 undefined *Enterobacter* spp. had GH33, while almost all the strains had GH20 and GH2 (Fig. 5F). Growth analysis of *E. coli* Nissle 1917 in ZMB1 with or without mucus, which was not one of the *E. coli* with GH33 expression in our genome analysis, confirmed the inability of this species to use mucus as the sole carbon source (Fig. 5G). These data suggest that commensal Proteobacteria are far less adept at degrading mucin than their gut microbiota counterparts.

**Figure 6:**
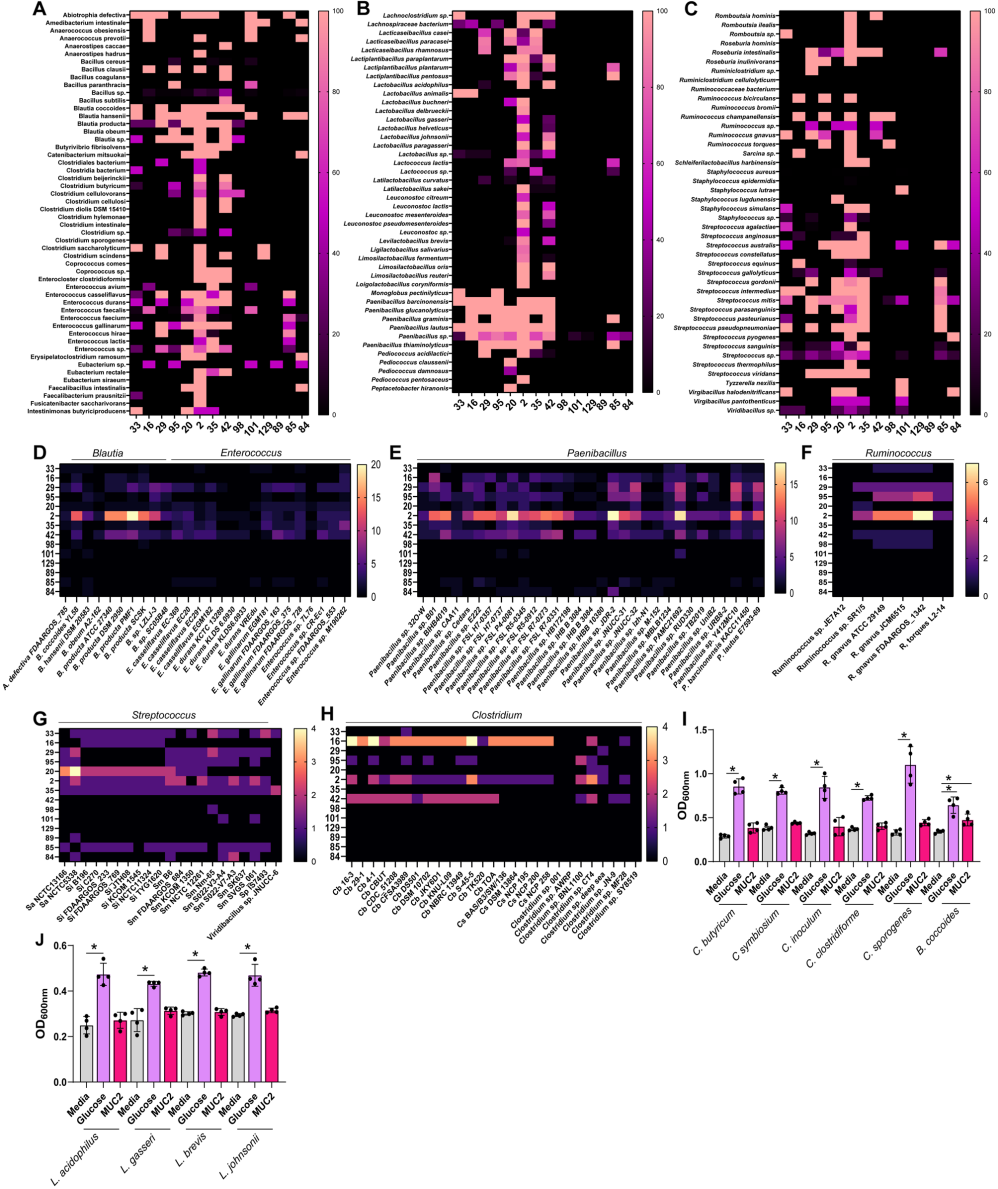
Mucin-related glycosyl hydrolase profiles in the Firmicutes phlyum. (**A**–**C**) Heat map of the Firmicutes genomes that have at least one gene copy of mucin-associated GH 33, 16, 29, 95, 20, 2, 35, 42, 98, 101, 129, 89, 85, and 84. Heat map showing the gene copy number of mucin-associated GHs in the strains of (**D**) *Abiotrophia defective*, *Blautia coccoides, B. hansenii, B. obeum, B. producta, Blautia* spp., *Enterococcus casseliflavus, E. durans, E. gallinarum*, and *Enterococcus* spp., (**E**) *Paenibacillus,* specifically *Paenibacillus* spp., *P barcinonensis and P. lautus,* (**D**) *Ruminococcus, including Ruminococcus* spp. *R. gnavus and R. torques,* (**F**) *Streptococcus, including S. australis (Sa), S. intermedius* (Si), *S. mitis* (Sm), *Streptococcus* spp. and *Viridibacillus* spp. (**G**) *Clostridium, including C. butyricum* (Cb), *C. sporogenes (*Cs), and *Clostridium* spp. (**H**,**I**) Growth analysis of *Clostridium butyricum* CB, *Clostridium symbiosum* ATCC 14940, *Clostridium inoculum* ATCC 14501, *Clostridium clostridiforme* ATCC 25532, and *Clostridium sporogenes* DSMZ 795 (**H**), as well as *Lactobacillus gasseri* ATCC 33323, *L. johnsonii* ATCC 33200, *L. brevis* ATCC 27305, *L. acidophilus* ATCC 4796 (**I**) in a chemically defined media ZMB1 lacking glucose (media control), with glucose (positive control), or lacking glucose and supplemented with 1 mg/mL porcine intestinal MUC2. Growth was measured by examining the optical density at 600 nm (OD_600nm_) after overnight incubation.

Finally, we examined the Firmicutes phylum and found that *Abiotrophia, Blautia, Enterococcus, Paenibacillus, Ruminococcus, Streptococcus,* and *Viridibacillus* species harbored several mucin-degrading GHs (Fig. 6A–C). The one genome of *Abiotrophia defectiva* had one gene copy of GH33 and 1–2 gene copies of GH29, GH20, GH2, GH35, GH101, and GH85. Within *Blautia*, *B. coccoides* and *B. hansenii* had one gene copy of GH33 and genes for GH29, GH95, GH20, GH2, GH101, GH85 and GH84 (Fig. 6D). In contrast, *B. obeum, B. producta* and undefined *Blautia* spp. had no gene copies of GH33, but did have variable gene copies (0–20) of GH16, GH29, GH20, GH2, GH35, and GH42, indicating the ability to remove fucose and galactose. Among the *Enterococcus* strains, only 1 of the 4 *E. casseliflavus* strains, 1 of the 4 *E. durans* strains, 2 of the 4 *E. gallinarum*, and 1 of the 4 undefined *Enterococcus* spp. possessed GH33. Variable numbers of gene copies were observed in GH29, GH95, GH20, GH2, GH35, GH42 and GH85. In *Paenibacillus* (Fig. 6E), we observed that *P. barcinonensis* and *P. lautus* genomes had one gene copy of GH33 and both genomes harbored GH16, GH29, GH95, GH2, and GH35, while *P. lautus* also had gene copies for GH20 and GH85. Of the 29 genomes of undefined *Paenibacillus* spp., we found that only 4 strains had GH33, but the majority of strains had gene copies of GH16, GH29, GH29, GH95, GH20, GH2, GH35 and GH42, suggesting that *Paenibacillus* spp. can remove fucose and galactose. We observed that all three *Ruminococcus gnavus* genomes had one gene copy of GH33, while undefined *Ruminococcus* spp. and *R. torques* did not harbor GH33 (Fig. 6F). Most *Ruminococcus* strains possessed GH29, GH85, GH2 and GH42. Among the streptococci, we found that all 8 *S. intermedius* genomes contained GH33, GH29, GH20, GH2, GH35, and GH85 (Fig. 6G). We also observed that 6 of the 9 S*. mitis* spp. had GH33 and most strains had gene copies of GH29, GH95, GH20, GH2, GH35 and GH85. Only one genome was available for *Viridibacillus* spp. and this genome had GH33 and GH35.

Pathogenic *Clostridium* spp., such *C. perfringens*, have previously been shown to degrade mucins^35^, but little information exists on mucin degradation by commensal *Clostridium* spp. Of the 14 *C. butryicum* genomes, we found that only one of the genomes harbored GH33 and none of the *C. sporogenes* or undefined *Clostridium* spp. possessed GH33 (Fig. 6H). Compared to other species, commensal *Clostridium* spp. had only a few mucin-associated GHs, including GH16, GH95, and GH42. These profiles suggest that commensal *Clostridium* spp. are unlikely to be involved in substantial mucin degradation. Based on our genome analysis, we predicted that commensal *Clostridium* spp. could not degrade intact mucus and use mucus to enhance growth. To address this question, we examined the growth of several *Clostridium* spp., including *C. butryicum* CB, *C. symbiosum* ATCC 14940, *C. inoculum* ATCC 14501, *C. clostridiforme* ATCC 25532, and *C. sporogenes* DSMZ 795 in media with or without mucus (Fig. 6I). Consistent with our analysis, none of the *Clostridium* spp. had enhanced growth with mucus. Our genome analysis indicated that *Blautia coccoides* possessed multiple GHs involved in mucin degradation and we predicted that this strain would be capable of using mucin glycans as the sole carbon source. Similar to our GH profile, we found that *B. coccoides* had statistically significant growth with mucus compared to media without mucus. Finally, we examined *Lactobacillus*, which according to our genome analysis only have 1–4 mucin-associated GHs and do not harbor GH33. We grew *Lactobacillus gasseri* ATCC 33323, *L. johnsonii* ATCC 33200, *L. brevis* ATCC 27305, and *L. acidophilus* ATCC 4796 in media with and without mucus and found that mucus did not enhance the growth of many *Lactobacillus* spp. (Fig. 6J). These data provide a comprehensive analysis of mucin-associated GH profiles within commensal gut microbes and highlight that only specific gut strains have mucin-degrading capacity.

## Discussion

To survive in the ever-changing environment of the gastrointestinal tract, gut microbes must be adept at foraging for nutrient sources. One way microbes deal with the varying availability of dietary carbohydrates is to forage glycans in the host mucus layer^3^. Mucin glycans are degraded by the sequential action of multiple mucin-associated GHs^30^. Sialic acid residues that terminate mucin glycans have been proposed to limit glycan degradation, thereby protecting the mucin glycan structure. Mucin glycans are also commonly terminated by fucose residues. As a result, mucin degrading microbes commonly encode sialidases and fucosidases to remove the terminal glycan structures, which then allow access to the extended core structures. Freed monosaccharides can then be used by the mucin-degrading bacteria themselves or scavenged by other bacteria^2,7^. Our comparative genomic approach has revealed that many gut microbes found in healthy individuals possess GH33 and other mucin-degrading GHs, indicating that these microbes have the capacity for extensive mucin degradation. Consistent with other findings, we found that *Akkermansia* harbored 9 different GH families and *A. muciniphila* ATCC BAA-835 was able to grow in a chemically defined medium with porcine intestinal mucus as the sole carbon source. We also identified several commensal bacteria with mucin-associated GH profiles and GHs numbers (≥ 8 GH families) similar to *Akkermansia*, including *Victavallales bacterium, Bifidobacterium bifidum, Streptomyces lividans, Blautia coccoides, B. hansenii, Bacteroides caccae, B. cellulosilyticus, B. dorei, B. fragilis, B. intestinalis, B. ovatus, B. thetaiotaomicron, B. uniformis, B. vulgatus, B. xylanisolvens, Fermentimonas caenicola, Parabacteroides distasonis, Phocaeicola coprophilus, P. dorei, P. vulgatus,* and *Paenibacillus lautus.* These genomic data suggest that several gut microbes may be able to completely degrade intestinal mucin glycans.

Two microbes we identified that appear to possess the ability for extensive mucin degradation are *Victavallales bacterium* and *Fermentimonas caenicola.* The family Victivallaceae has only two cultured representatives: *Victivallis vadensis* strain CelloT and the uncharacterized strain NML 080035^36^. These microbes are Gram-negative and anaerobic. There are also 16S rRNA gene sequences from uncultured Victivallaceae. Culturable *V. vadensis* can use galactose as a primary carbon source^36^. In our genome analysis, we found that *Victivallales bacterium* possessed GH33, GH16, GH29, GH95, GH20, GH2, GH35, GH42, GH89 and GH129. Several of these glycosyl hydrolases are galactosidases (GH2, GH20, GH35, and GH42). As a result, we predict that *Victivallales bacterium* may cleave mucin galactose residues to use as a carbon source. *F. caenicola* was isolated from the stool of a healthy Senegalese child as part of a study aiming at cultivating human gut microbes^37^. *F. caenicola* is Gram-negative, facultatively anaerobic bacillus. Beye et al*.* found using an API 50 CH strip that *F. caenicola* also grows with galactose^37^. In our genome analysis, we found that *F. caenicola* harbors several galactosidases (GH2, GH20, and GH42), suggesting that removal of galactose from glycan core structures could promote *F. caenicola* growth. Our analysis also identified that the *F. caenicola* genome had gene copies of GH29, which contains a fucosidase. Interestingly, Beye et al*.* found that *F. caenicola* was unable to grow with d-fucose or l-fucose. Other microbes, like *B. bifidum*, have been shown to cleave fucose and cross-feed other bacteria, like *Eubacterium hallii*, which cannot degrade complex glycans^38^. It is possible that fucose release by *F. caenicola* may promote the growth of other microbes. Although there are no studies examining growth of *Victavallales bacterium* or *F. caenicola* with other mucin-related sugars or intact mucins, based on the GH profile, we predict that these microbes could use intact mucins and potentially use other mucin glycan sugars as carbon sources.

Although we focused on microbes with GH profiles indicative of more complete mucin-degradation, it is well known that microbes can act in concert to break down glycan structures. In pioneering studies in the 1980s, Hoskins et al*.* examined fecal bacteria grown in mucin-based medium and found that 1% of the microbiota was able to use mucin as a carbon source, including the genera *Bifidobacterium* and *Ruminococcus*^39,40^. Recent in silico analysis, which is not reliant on culturing techniques, has demonstrated that up to 86% of the human gut microbiota encode genes for cleavage of mucin glycans^23^. We also found that 62% of all microbes and 83% of human gut microbes in the CAZy database encode genes for mucin-degradation. These studies, as well as our own, have found that only specific bacterial species have a sufficient repertoire of enzymes to disassemble complex mucin glycans and that the complete degradation of mucin often requires the action of several bacteria. Our analysis reveals that many bacteria possess multiple gene copies of GHs targeting internal glycans. These findings suggest that mucolytic bacteria with GH33 may initiate glycan break-down and then the less-specialized bacteria with internal glycan GHs can participate in degradation.

Based on our studies, we believe that the core GH-ome for mucin degradation includes GH33, 29, 95, and 20/35. More extensive degradation of internal glycans incorporates GH84/85/89 and 101. Our genome analysis suggests that mucin degrading microbes possess > 4 mucin-associated GHs. Additionally, microbes that extensively degrade mucin, like *A. muciniphila*, *B. bifidum*, and *B. thetaiotaomicron*, possess > 9 mucin-associated GHs. Despite the fact that many commensal microbes are not capable of extensive mucin degradation, the GH profile of bacteria such as *Clostridium* indicates that they could contribute to degradation when paired with another bacteria. For example, if *A. muciniphila* removes sialic acid, several *Clostridium* species could remove fucose with GH95. After *A. muciniphila* removes N-acetyl-glucosamine, almost all *Clostridium* could remove galactose with GH2 or GH42. These cross-feeding events likely occur in vivo and contribute to the health of the mucus layer. Future studies using mucus cross-feeding will likely shed light into the complex interplay because mucin-degrading microbes.

Since mucin glycan degradation disrupts the first protection of the host mucus layer, host glycan foraging by mucolytic bacteria is commonly considered an initial stage in pathogenesis. While this notion likely only holds true for excessive mucin degradation, many consider mucin-glycan break-down to be a normal process and a key strategy for mucus-associated microbes. Given the continuous turnover of the epithelial cells and mucus in the human gastrointestinal tract, mucin degradation by commensal gut microbes is not likely to contribute to barrier dysfunction. Additionally, the capacity to degrade mucin is particularly important for early colonizers of the gut. Infants are commonly colonized with mucin degrading *Bifidobacterium bifidum, B. longum* subsp. *infantis,* and *B. breve*^41–44^. One study in Sweden identified the establishment of mucin-degrading bacteria during the first months of life^44^. We speculate that mucin glycan degradation gives colonizing microbes an advantage after the termination of breast milk and allows them to exert their beneficial influence on gut homeostasis.

One potential limitation of this work is that it is difficult to predict the exact specificity of a CAZyme based on family membership^45^. However, substrate categories can be broadly inferred within the CAZy families, even if the precise specificity of each protein in the family is challenging to predict^3^. An advantage of this type of genome analysis is that it does not require complex culturing, which can be challenging since many intestinal microbes are not able to grow in classical laboratory media. Genomic strategies have now been widely applied and are bringing new information about the diversity and function of human gut microbiota. Our genomic characterization has shed light on commensal gut species with mucin-degrading properties. We believe this work enhances the foundation for examining mucin-degradation within the human intestine.

## References

[1] Corfield AP, Wagner SA, Clamp JR, Kriaris MS, Hoskins LC (1992). Mucin degradation in the human colon: Production of sialidase, sialate O-acetylesterase, N-acetylneuraminate lyase, arylesterase, and glycosulfatase activities by strains of fecal bacteria. Infect. Immun..

[2] Bell A, Juge N (2021). Mucosal glycan degradation of the host by the gut microbiota. Glycobiology.

[3] Tailford LE, Crost EH, Kavanaugh D, Juge N (2015). Mucin glycan foraging in the human gut microbiome. Front. Genet..

[4] McGuckin MA, Linden SK, Sutton P, Florin TH (2011). Mucin dynamics and enteric pathogens. Nat. Rev. Microbiol..

[5] McDole JR (2012). Goblet cells deliver luminal antigen to CD103+ dendritic cells in the small intestine. Nature.

[6] Aihara E, Engevik KA, Montrose MH (2017). Trefoil factor peptides and gastrointestinal function. Annu. Rev. Physiol..

[7] Marcobal A, Southwick AM, Earle KA, Sonnenburg JL (2013). A refined palate: Bacterial consumption of host glycans in the gut. Glycobiology.

[8] Fang J (2021). Slimy partners: The mucus barrier and gut microbiome in ulcerative colitis. Exp. Mol. Med..

[9] Crouch LI (2020). Prominent members of the human gut microbiota express endo-acting O-glycanases to initiate mucin breakdown. Nat. Commun..

[10] Derrien M, Vaughan EE, Plugge CM, de Vos WM (2004). *Akkermansia muciniphila* gen. nov., sp. nov., a human intestinal mucin-degrading bacterium. Int. J. Syst. Evol. Microbiol..

[11] Sonnenburg JL (2005). Glycan foraging in vivo by an intestine-adapted bacterial symbiont. Science.

[12] Desai MS (2016). A dietary fiber-deprived gut microbiota degrades the colonic mucus barrier and enhances pathogen susceptibility. Cell.

[13] Pudlo NA (2015). Symbiotic human gut bacteria with variable metabolic priorities for host mucosal glycans. MBio.

[14] Martens EC, Chiang HC, Gordon JI (2008). Mucosal glycan foraging enhances fitness and transmission of a saccharolytic human gut bacterial symbiont. Cell Host Microbe.

[15] Comstock LE (2009). Importance of glycans to the host-bacteroides mutualism in the mammalian intestine. Cell Host Microbe.

[16] Png CW (2010). Mucolytic bacteria with increased prevalence in IBD mucosa augment in vitro utilization of mucin by other bacteria. Am. J. Gastroenterol..

[17] Kiyohara M (2012). alpha-N-acetylgalactosaminidase from infant-associated bifidobacteria belonging to novel glycoside hydrolase family 129 is implicated in alternative mucin degradation pathway. J. Biol. Chem..

[18] Bell A (2019). Elucidation of a sialic acid metabolism pathway in mucus-foraging *Ruminococcus gnavus* unravels mechanisms of bacterial adaptation to the gut. Nat. Microbiol..

[19] Crost EH (2013). Utilisation of mucin glycans by the human gut symbiont *Ruminococcus gnavus* is strain-dependent. PLoS ONE.

[20] Ruas-Madiedo P, Gueimonde M, Fernandez-Garcia M, de los Reyes-Gavilan CG, Margolles A (2008). Mucin degradation by Bifidobacterium strains isolated from the human intestinal microbiota. Appl. Environ. Microbiol..

[21] Salyers AA, West SE, Vercellotti JR, Wilkins TD (1977). Fermentation of mucins and plant polysaccharides by anaerobic bacteria from the human colon. Appl. Environ. Microbiol..

[22] Turroni F (2010). Genome analysis of *Bifidobacterium bifidum* PRL2010 reveals metabolic pathways for host-derived glycan foraging. Proc. Natl. Acad. Sci. USA.

[23] Ravcheev DA, Thiele I (2017). Comparative genomic analysis of the human gut microbiome reveals a broad distribution of metabolic pathways for the degradation of host-synthetized mucin glycans and utilization of mucin-derived monosaccharides. Front. Genet..

[24] Raimondi S, Musmeci E, Candeliere F, Amaretti A, Rossi M (2021). Identification of mucin degraders of the human gut microbiota. Sci. Rep..

[25] Trastoy B, Naegeli A, Anso I, Sjogren J, Guerin ME (2020). Structural basis of mammalian mucin processing by the human gut O-glycopeptidase OgpA from *Akkermansia muciniphila*. Nat. Commun..

[26] Bjursell MK, Martens EC, Gordon JI (2006). Functional genomic and metabolic studies of the adaptations of a prominent adult human gut symbiont, *Bacteroides thetaiotaomicron*, to the suckling period. J. Biol. Chem..

[27] Zhang G, Mills DA, Block DE (2009). Development of chemically defined media supporting high-cell-density growth of lactococci, enterococci, and streptococci. Appl. Environ. Microbiol..

[28] Cantarel BL (2009). the carbohydrate-active enzymes database (CAZy): An expert resource for glycogenomics. Nucleic Acids Res..

[29] El Kaoutari A, Armougom F, Gordon JI, Raoult D, Henrissat B (2013). The abundance and variety of carbohydrate-active enzymes in the human gut microbiota. Nat. Rev. Microbiol..

[30] Lombard V, Golaconda Ramulu H, Drula E, Coutinho PM, Henrissat B (2014). The carbohydrate-active enzymes database (CAZy) in 2013. Nucleic Acids Res..

[31] Park BH, Karpinets TV, Syed MH, Leuze MR, Uberbacher EC (2010). CAZymes Analysis Toolkit (CAT): Web service for searching and analyzing carbohydrate-active enzymes in a newly sequenced organism using CAZy database. Glycobiology.

[32] Henrissat B, Bairoch A (1993). New families in the classification of glycosyl hydrolases based on amino acid sequence similarities. Biochem. J..

[33] Henrissat B (1991). A classification of glycosyl hydrolases based on amino acid sequence similarities. Biochem. J..

[34] Chen IA (2021). The IMG/M data management and analysis system v.6.0: New tools and advanced capabilities. Nucleic Acids Res..

[35] Low KE, Smith SP, Abbott DW, Boraston AB (2021). The glycoconjugate-degrading enzymes of *Clostridium perfringens*: Tailored catalysts for breaching the intestinal mucus barrier. Glycobiology.

[36] Plugge, C. M. & Zoetendal, E. G. In *The Prokaryotes: Other Major Lineages of Bacteria and The Archaea* (eds Rosenberg, E. *et al.*) 1019–1021 (Springer, 2014).

[37] Beye M (2018). Draft genome sequence of Fermentimonas caenicola strain SIT8, isolated from the human gut. Stand Genomic Sci..

[38] Bunesova V, Lacroix C, Schwab C (2018). Mucin cross-feeding of infant Bifidobacteria and *Eubacterium hallii*. Microb. Ecol..

[39] Miller RS, Hoskins LC (1981). Mucin degradation in human colon ecosystems. Fecal population densities of mucin-degrading bacteria estimated by a "most probable number" method. Gastroenterology.

[40] Hoskins LC, Boulding ET (1981). Mucin degradation in human colon ecosystems. Evidence for the existence and role of bacterial subpopulations producing glycosidases as extracellular enzymes. J. Clin. Investig..

[41] Lim ES (2015). Early life dynamics of the human gut virome and bacterial microbiome in infants. Nat. Med..

[42] Backhed F (2015). Dynamics and stabilization of the human gut microbiome during the first year of life. Cell Host Microbe.

[43] Makino H (2013). Mother-to-infant transmission of intestinal bifidobacterial strains has an impact on the early development of vaginally delivered infant's microbiota. PLoS ONE.

[44] Midtvedt AC, Carlstedt-Duke B, Midtvedt T (1994). Establishment of a mucin-degrading intestinal microflora during the first two years of human life. J. Pediatr. Gastroenterol. Nutr..

[45] Cantarel BL, Lombard V, Henrissat B (2012). Complex carbohydrate utilization by the healthy human microbiome. PLoS ONE.

